# Short-term therapy with R568 ameliorated secondary hyperparathyroidism but does not prevent aortic valve calcification in uremic rats

**DOI:** 10.3389/fneph.2024.1385705

**Published:** 2024-08-06

**Authors:** Asmahan Abu-Snieneh, Irina Gurt, Suzan Abedat, Chaim Lotan, Michael Glikson, Mony Shuvy

**Affiliations:** ^1^ Heart Institute, Cardiovascular Research Center, Hadassah-Hebrew University Medical Center, Jerusalem, Israel; ^2^ Jesselson Integrated Heart Center, Shaare Zedek Medical Center and Faculty of Medicine, Hebrew University, Jerusalem, Israel

**Keywords:** aortic valve calcification, chronic kidney disease, phosphate, R568, animal model

## Abstract

**Introduction:**

Renal failure associated aortic valve calcification (AVC) is the result of hyperphosphatemia and hyperparathyroidism. Calcimimetics is an effective tool for management of secondary hyperparathyroidism. Our goal was to evaluate the effect of the medical intervention with calcimimetic R568 on the AVC process.

**Methods and results:**

The experimental design consisted of administering a uremia-inducing phosphate-enriched diet to rats for six weeks. Rats received a daily R568 injection at different times. Biochemical analysis demonstrated increased urea (34.72 ± 3.57 *vs*. 5.18 ± 0.15 mmol/L, *p*<0.05) and creatinine (293.93 ± 79.6 *vs*. 12.82 ± 1.56 µmol/L, *p*<0.05). R568 treatment markedly reduced parathyroid hormone (PTH) levels in both treated groups (192.63 ± 26.85, 301.23 ± 101.79 *vs*. 3570 ± 986.63 pg/mL, *p*<0.05), with no impact on serum calcium and phosphate. von Kossa staining showed increase in AVC in uremic rats compared to control (1409 ± 159.5 *vs*. 27.33 ± 25.83, *p*<0.05). AVC was not affected by R568 in both groups (3343 ± 2462, 1593 ± 792 *vs*. 1409 ± 159.5, NS). Similarly, the inflammatory marker CD68 was elevated in uremic rats (15592 ± 3792 *vs*. 181.8 ± 15.29, *p*<0.01), and was not influenced by R568 treatment (8453 ± 818.5, 9318 ± 2232 *vs*. 15592 ± 3792, NS). Runt-related transcription factor 2 (Runx2), the regulator of osteoblast differentiation, was upregulated in uremic rats (23186 ± 9226 *vs*. 3184 ± 2495), that accompanied by elevated levels of Osteopontin (158395 ± 45911 *vs*. 237.7 ± 81.5, *p*<0.05) and Osteocalcin (22203 ± 8525 *vs*. 489.7 ± 200.6, *p*<0.05). R568 had no impact on osteoblastic markers (Runx2: 21743 ± 3193, 23004 ± 10871 *vs*. 23186 ± 9226, NS; osteopontin: 57680 ± 19522, 137116 ± 60103 *vs*. 158395 ± 45911, NS; osteocalcin: 10496 ± 5429, 8522 ± 5031 *vs*. 22203 ± 8525, NS).

**Conclusion:**

In an adenine-induced uremic rat model, we showed that short-term R568 therapy had no effect on AVC. Treatment with R568 decreased PTH levels but had no effect on high phosphate levels. Regression of AVC necessitates not only a decrease in PTH levels, but also a decline in phosphate levels. To achieve improved outcomes, it is advisable to consider administering a combination of R568 with other medications, such as calcium supplements or phosphate binders. Additional studies are required for further evaluation of the potential treatment of chronic kidney disease (CKD)-associated AVC.

## Introduction

Aortic stenosis (AS) is the most common reason for aortic valve replacement in the United States and Europe (1). The two most notable characteristics of AS are aortic valve calcification (AVC) and valve thickening (2, 3). Currently, no therapy can halt AVC progression, therefore valve replacement is the sole treatment option for patients with severe AS.

Over the last decade, numerous studies have shed light on the active and regulated process of AVC. These studies have revealed that AVC involves inflammation (4), alterations in extracellular matrix, and the transformation of aortic valve interstitial cells (AVICs) into osteoblasts, which are responsible for bone formation bone-producing cells (5). The culmination of these processes leads to the calcification of valve leaflets, ultimately resulting in valve stenosis (6).

Renal failure (RF) has emerged as a significant risk factor for AVC, with approximately 40% of patients suffering from end-stage renal disease developing AVC and AS (7). In the last two decades, population-based studies demonstrated that between 28 and 85% of chronic kidney disease (CKD) patients developed AVC, whereas 6 – 13% of patients on hemodialysis displayed severe AS. This is a significantly higher prevalence compared to what is found in the general population, where approximately 25% of individuals over 65 have AVC (8). The prevalence and extent of AVC in this population is attributed to mineral metabolism such as hyperphosphatemia and hormonal changes that are likely to promote AVC development and progression.

Secondary hyperparathyroidism (SHPT), commonly develops during stages 3 and 4 of CKD, may lead to cardiovascular calcifications by other mechanisms including an impaired effect of parathyroid hormone (PTH), and a decreased calcium-sensing receptor (CaR) expression on cardiovascular structures (9). Unregulated mineral metabolism, increased levels of phosphorus and the calcium-phosphorus product (Ca×P), contribute to the onset of vascular calcification. Elevated phosphate and PTH levels in CKD are associated with increased vascular calcification, arterial stiffness, and the risk of cardiovascular events. SHPT triggers the release of phosphate and calcium from bones, leading to increased ectopic calcification. Elevated levels of PTH are correlated to AVC in RF, observed in both animal studies and human subjects (10). Vitamin D analogs are often used to treat hyperparathyroidism; however, this treatment can lead to elevated serum levels of both calcium and phosphate, consequently exacerbating vascular calcification (11).

In CKD, the kidneys’ capacity to remove phosphorus from the blood diminishes, leading to elevated serum phosphorus levels. Prolonged hypocalcemia, hyperphosphatemia, reduced calcitriol, and heightened fibroblast growth factor-23 (FGF-23) levels all contribute to increased synthesis and release of PTH. Phosphate retention and elevated FGF-23 levels decrease calcitriol production, thereby causing an additional indirect pathway for elevated PTH synthesis (12–14). The persistence of these stimuli results in a gradual decline in the expression of CaR in the parathyroid glands, reducing the sensitivity of these glands to changes in serum calcium levels and calcitriol, which may indicate irreversible parathyroid gland hyperplasia.

CaR, a class C G-protein-coupled receptor located on the surface of parathyroid chief cells, is pivotal in calcium-mediated signaling within these cells, regulating both PTH secretion and synthesis (15). This receptor has significant implications for maintaining proper calcium balance by sensing changes in extracellular ionized calcium concentration, thus being an attractive target for the development of treatment compounds, such as calcimimetics. Calcimimetics alter the structural conformation of the CaR and stereo-selectively enhance its sensitivity to Ca^2+^. Phenylalkylamines, like NPS R-568, directly affect the CaR. As far as investigated, the impact of these compounds on the function of parathyroid cells mirrors that of extracellular Ca^2+^; specifically, they induce rises in [Ca^2+^]i by mobilizing intracellular Ca^2+^ and facilitating extracellular Ca^2+^ influx, consequently inhibiting PTH secretion. Initial findings suggest that these phenylalkylamine compounds also hinder β-adrenergic receptor-triggered elevation in cyclic adenosine monophosphate (cAMP) formation in a manner sensitive to pertussis toxin, akin to extracellular Ca^2+^ (16). In animal studies, administration of the calcimimetic R568 provoked a rapid and dose-dependent decrease in serum PTH and calcium concentrations (17–19). Cinacalcet (CINA) has emerged as a powerful tool in the management of SHPT. Notably, recent studies shown that by suppressing PTH, CINA can effectively attenuate vascular remodeling and calcification in models of CKD (20). Animal models have demonstrated remarkable success in preventing or reversing vascular calcification with this class of compounds (21–25).

Based on these findings, we sought to evaluate if AVC process is influenced by medical intervention with R568, which regulates the level of PTH that are affected by abnormal phosphate levels. We previously described our CKD model for AVC that is based on a high-adenine (0.75%) and phosphate-enriched (1.5%) diet, causing significant renal insufficiency and AVC (26). High-adenine diet induces severe tubular injury characterized by extensive renal cystic dilatation and deposition of 8-dihydroxyadenine. Adenine accumulation in the renal proximal tubules led to elevated serum creatinine, phosphate, and PTH within 2-3 weeks. In the present study, we focused on the pharmacological effect of R568 on uremic rats: we administered the drug at various points and evaluated its impact at various stages of AVC. We aimed to assess if there is a certain stage in which pharmaceutical interventions.

## Materials and methods

### Animal model for CKD-associated valve disease

**Figure 1:**
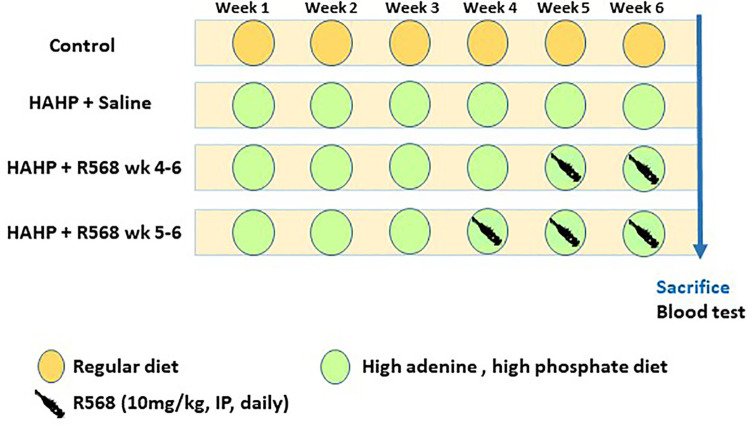
Study protocol. Twenty-two eight-week-old male Sprague-Dawley rats were used for the study. After 6 weeks, all animals were euthanized at the same time point. Blood tests were performed at the end point.

Eight-week-old male Sprague-Dawley rats (n=22, 250 g) were purchased from Envigo RMS (Israel) Ltd. Animals were housed under specific pathogen-free conditions with a 12-hour light/dark cycle (lights on 07:00–19:00), in an environmentally-controlled room at a temperature of 23 ± 2°C and humidity of 55% ± 10%. Animals were randomly divided into four groups: six rats (control group) received regular diet and three groups received high-adenine (0.75%) phosphate-enriched (1.5%) diet (HAHP diet). Four rats received saline intraperitoneal (IP) injection (HAHP + saline); six rats received daily 10 mg/kg of R568 (calcimimetic) IP injection during the last three weeks of the study (HAHP + R568 week 4-6); six rats received 10 mg/kg of R568 IP injection during the last two weeks of the study (HAHP + R568 week 5-6) (**Figure 1**). After six weeks, all animals were euthanized by anesthesia with 5% isoflurane in oxygen (4 L/min). Blood was collected by direct heart puncture and aortic valves were immediately harvested and kept in -80°C (for protein extraction) or in 80% ethanol (for paraffin section). The study was approved by the Hebrew University Ethics Committee for the Care and Use of Laboratory Animals (permit no. 146944, approval number MD-15646-5). The study was conducted in accordance with ARRIVE Guidelines.

### Effect of diet on biochemical profile

Plasma was analyzed for creatinine, urea, calcium and phosphate using the VITROS 5.1 FS chemistry system (Ortho-Clinical Diagnostics; Johnson & Johnson, Rochester, NY). Plasma PTH was measured by PTH ELISA 1-84 assay (Quidel; Athens, Ohio, USA).

### Von Kossa staining

Serial cross-sections of the valve were stained with von Kossa to assess the structure and mineral deposits. They were incubated for 90 minutes with 5% silver nitrate (Sigma-Aldrich) under a 60-watt white lamp. After washing with deionized water, sections were incubated for five minutes with 5% sodium thiosulfate (Sigma-Aldrich) to remove unreacted silver. Images were obtained with an EVOS M5000 microscope and analyzed with ImageJ software. Calcified areas were normalized to valve unit area, as previously reported (27).

### Immunofluorescence staining

Rat aortic valves were harvested upon sacrifice, fixed with 4% buffered formaldehyde and embedded in paraffin. For antigen retrieval, 5-µm sections were incubated 10 minutes at 60°C with Antigen Unmasking Retrieval solution (H-3300, Vector Laboratories), washed with phosphate-buffered saline (PBS), blocked with 3% bovine serum albumin (BSA), and incubated with antibodies to osteocalcin, Runx-2, osteopontin, and CD68 (Santa Cruz Biotechnology Inc.; CA, USA) at 4°C overnight. After washing with PBS, the sections were incubated for one hour with Cy5-conjugated secondary antibody at room temperature. Nuclear counterstaining was performed with 4,6-diamidino-2-phenylindole (DAPI). Images were obtained with an EVOS M5000 fluorescent microscope. Four random areas from different parts of the valves were analyzed by Image Pro-Plus version 7.0 (Media Cybernetics; Rockville, MD, USA) software.

### Western blot analysis

Aortic valves were suspended in RIPA buffer (#89900, Thermo Scientific) with protease inhibitor cocktail (#87786, Thermo Scientific). Twelve micrograms of total protein was analyzed by Western blot. To verify, osteopontin and RUNX2, with nearly overlapping molecular weights of 55 kDa and 65 kDa respectively, were loaded onto separate gels.

Antibodies for immunoblotting: anti-Runx-2 (sc-390351, Santa-Cruz Biotechnology Inc.; CA, USA, dilution 1:100), anti-osteopontin (Ab11503, Abcam; Cambridge, UK, dilution 1:300), anti-actin (Cf-0869100, MP Biomedical, dilution 1:500), and secondary antibodies (Jackson Laboratories; Bar Harbor, ME, USA). Band intensity quantitation was done by using ImageJ software.

### Statistical analysis

Results are presented as means ± standard deviation (SD). Nonparametric Kruskal-Wallis one-way analysis followed by Dunn’s *post-hoc* correction was performed with Prism 9 (GraphPad Software, Inc., San Diego, CA, USA). All performed tests were two-tailed, and a difference of *p<*0.05 was considered statistically significant.

## Results

### The effect of R568 treatment on kidney and parathyroid function

Rats in all three groups that received HAHP diet developed kidney dysfunction, which was indicated by increased serum levels of creatinine and urea (creatinine: 293.93 ± 79.6, 221.15 ± 35.4, 194.48 ± 13.5 *vs*. 12.82 ± 1.56 µmol/L, *p*<0.05 *vs*. control; urea: 34.72 ± 3.57, 38.7 ± 2.9, 38.9 ± 2.3 *vs*. 5.18 ± 0.15 mmol/L, respectively, *p*<0.05 *vs*. control). We observed increased serum levels of phosphate and decreased serum levels of calcium in uremic animals, confirming impaired mineral metabolism (**Table 1**). Serum PTH levels were markedly elevated in uremic rats, and R568 administration abolished SHPT in both treated groups (192.63 ± 26.85, 301.23 ± 101.79 *vs*. 3570 ± 986.63 pg/mL, *p*<0.05 *vs*. HAHP + saline). Treatment with R568 has no significant effect on serum calcium and phosphate levels in uremic rats (Ca: 1.8 ± 0.06, 1.9 ± 0.03 *vs*. 2.18 ± 0.28; Phos: 6.02 ± 0.53, 5.09 ± 0.4 *vs*. 4.97 ± 0.5).

**Table 1:** Biochemical evaluation. Biochemical profiles of the phosphate-enriched uremic diet group, phosphate-enriched uremic group with 3 weeks of R568 treatment, phosphate-enriched uremic group with 2 weeks of R568 treatment, and a control group that was fed a regular diet.

	Creatinine (µmol/L)	BUN (mmol/L)	Ca (mmol/L)	Phos (mmol/L)	PTH (pg/ml)
Control	12.82 ± 1.56	5.18 ± 0.15	2.65 ± 0.04	3.31 ± 0.11	587.9 ± 55.42
HAHP + Saline	293.93 ± 79.6 ^b^	34.72 ± 3.57	2.18 ± 0.28	4.97 ± 0.51	3570 ± 986.63
HAHP + CINA w 4-6	221.15 ± 35.4 ^a^	38.7 ± 2.9 ^a^	1.8 ± 0.06 ^b^	6.02 ± 0.53 ^b^	192.63 ± 26.85 ^d^
HAHP + CINA w 5-6	194.48 ± 13.5 ^a^	38.9 ± 2.3 ^a^	1.9 ± 0.03 ^a^	5.09 ± 0.4 ^a^	301.23 ± 110.79 ^c^

Results are mean ± SE, analized by nonparametric Kruskal-Wallis one-way analysis followed by Dunn’s posthoc comparisons; ^a^ P< 0.05 vs control; ^b^ P< 0.01 vs control; ^c^ P< 0.05 vs HAHP + Saline; ^d^ P< 0.01 vs HAHP + Saline.

### The effect of R568 treatment on aortic valve calcification

**Figure 2:**
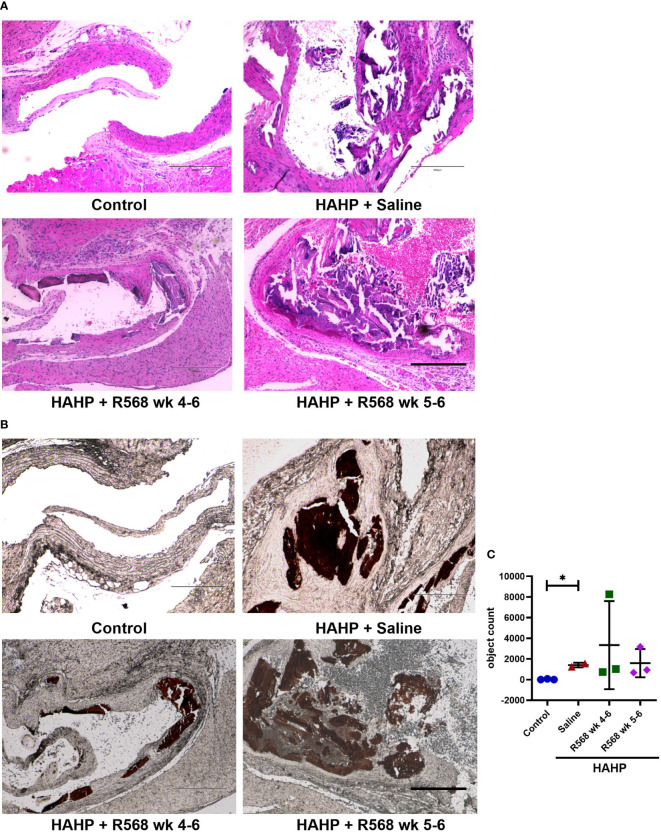
The effect of R568 treatment on aortic valve calcification. **(A)** Representative images of Hematoxylin-eosin (H&E) staining of aortic valve sections obtained from different groups (n=2-3 rats/group). The images were taken at 10x magnification, bar: 300µm. **(B, C)** Representative images **(B)** and quantitation **(C)** of von Kossa staining of aortic valve section obtained from different groups (n=2-3 rats/group). Valvular calcification was observed by von Kossa stain (brown color) in uremic rats. The images were taken at 10x magnification, bar: 300µm. Data are means ± SD, analyzed by Kruskal-Wallis one-way analysis followed by Dunn’s *post hoc* correction, *p<0.05 vs. control.

The HAHP diet led to a significant increase in AVC in all three groups, as evidenced by von Kossa staining (1409 ± 159.5 *vs*. 27.33 ± 25.83, *p*<0.05 *vs*. control). However, it does not seem that R568 treatment had any effect on calcification (**Figure 2**), and there were no significant changes in AVC in valves of rats in both treated groups (3343 ± 2462, 1593 ± 792 *vs*. 1409 ± 159.5).

### The effect of R568 treatment on osteoblastic marker expression

**Figure 3:**
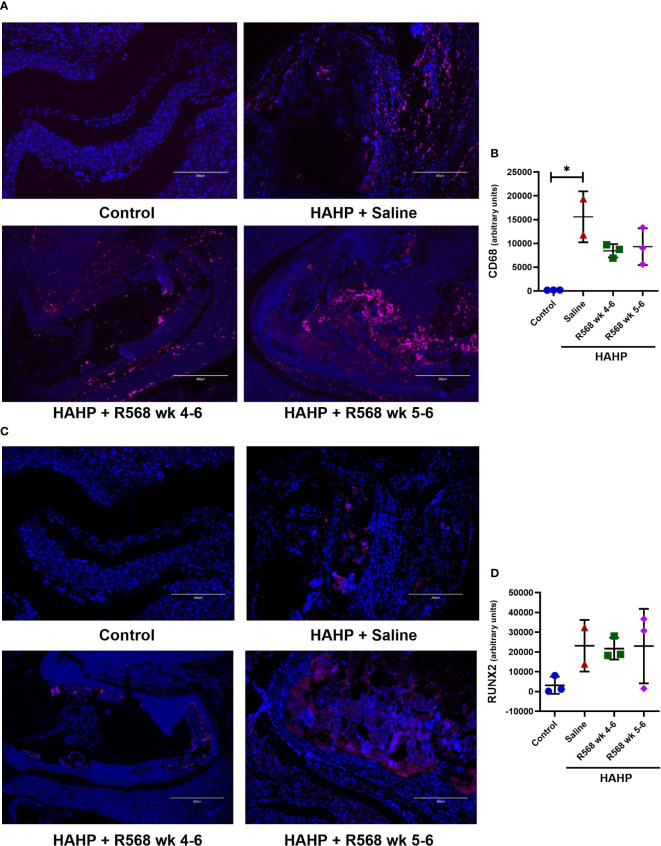
The effect of R568 treatment on early osteoblast markers expression in aortic valve. **(A, B)** Representative images **(A)** and quantification **(B)** of CD68 staining of aortic valve sections obtained from different groups (n=2-3 rats/group). The positive immunofluorescence for CD68 (pink) was observed; 4,6-diamidino-2-phenylindole (blue) stained nuclei. The images were taken at 10x magnification, bar: 300µm. **(C, D)** Representative images **(C)** and quantification **(D)** of Runx2 staining of aortic valve sections obtained from different groups (n=2-3 rats/group). The positive immunofluorescence for Runx2 (pink) was observed; 4,6-diamidino-2-phenylindole (blue) stained nuclei. The images were taken at 10x magnification, bar: 300µm. Data are means ± SD, analyzed by Kruskal-Wallis one-way analysis followed by Dunn’s *post hoc* correction, *p<0.05 vs. control.

**Figure 4:**
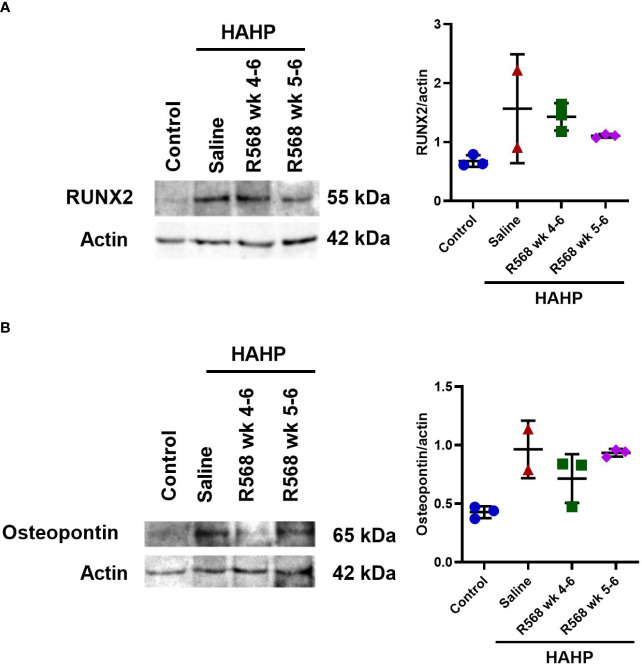
The effect of R568 treatment on osteoblast markers protein expression in aortic valve. Representative images and quantitation of **(A)** Runx2 and **(B)** osteopontin western blot of aortic valve sections obtained from different groups (n=2-3 rats/group). Data are means ± SD.

In aortic valves of uremic rats, increased CD68 appearance was found compared to controls (15592 ± 3792 *vs*. 181.8 ± 15.29, *p*<0.01 *vs*. control); moreover, in both R568-treated groups no significant difference with untreated uremic animals was found (8453 ± 818.5, 9318 ± 2232 *vs*. 15592 ± 3792) (**Figures 3A, B**). Runt-related transcription factor 2 (Runx2), the key regulator of osteoblastogenesis, presented seven-fold more in aortic valves of uremic rats compared to controls (**Figures 3C, D**). Like for CD68, R568 treatment had no effect on Runx2 immunofluorescence in aortic valves of treated animals (21743 ± 3193, 23004 ± 10871 *vs*. 23186 ± 9226). We verified Runx2 immunofluorescence results by western blot, showing again no change in protein levels after R568 (**Figure 4A**). Raised Runx2 levels promote differentiation of mesenchymal cells to osteoblast and cause expression of osteoblast markers.

**Figure 5:**
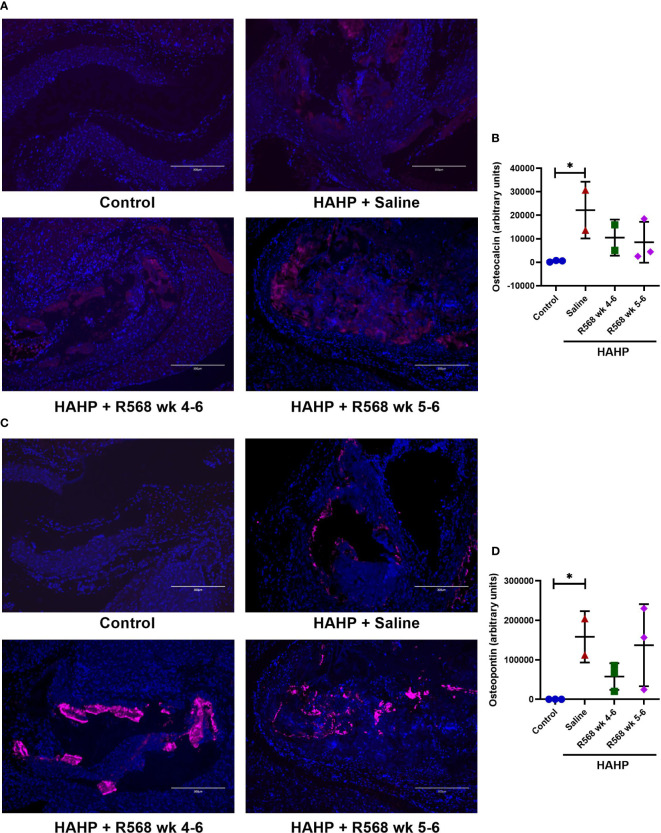
The effect of R568 treatment on late osteoblast markers expression in aortic valve. **(A, B)** Representative images **(A)** and quantification **(B)** of osteocalcin staining of aortic valve sections obtained from different groups (n=2-3 rats/group). The positive immunofluorescence for osteocalcin (pink) was observed; 4,6-diamidino-2-phenylindole (blue) stained nuclei. The images were taken at 10x magnification, bar: 300µm. **(C, D)** Representative images **(C)** and quantification **(D)** of osteopontin of aortic valve sections obtained from different groups (n=2-3 rats/group). The positive immunofluorescence for osteopontin (pink) was observed; 4,6-diamidino-2-phenylindole (blue) stained nuclei. The images were taken at 10x magnification, bar: 300µm. Data are means ± SD, analyzed by Kruskal-Wallis one-way analysis followed by Dunn’s *post hoc* correction, *p<0.05 vs. control.

We observed significantly increased immunofluorescence of Osteopontin and Osteocalcin in valves of rats that received a HAHP diet compared to controls group (osteopontin: 158395 ± 45911 *vs*. 237.7 ± 81.5, *p*<0.05 *vs*. control; osteocalcin: 22203 ± 8525 *vs*. 489.7 ± 200.6, *p*<0.05 *vs*. control). As expected, no difference between R568- and saline-treated animals was detected (osteopontin: 57680 ± 19522, 137116 ± 60103 *vs*. 158395 ± 45911; osteocalcin: 10496 ± 5429, 8522 ± 5031 *vs*. 22203 ± 8525) (**Figure 5**).

We confirmed Osteopontin immunofluorescence results by western blot (**Figure 4B**). Like Runx2, R568 treatment had no effect on increased Osteopontin protein levels in both treated groups.

## Discussion

In the present study, we demonstrated that short-term R568 treatment had no effect on AVC in an adenine-induced uremic rat model. Similar to our previous work (28), a high adenine diet induced RF in rats, validated by elevated creatinine and blood urea nitrogen (BUN) levels. Although R568 treatment markedly reduced serum PTH levels, it had no impact on elevated phosphate levels in uremic rats. It is noteworthy that serum phosphate levels are significantly associated with AVC prevalence in CKD patents (29). As we showed, phosphate is essential for the initiation of the calcification process and elevated PTH level had no promote to AVC without increased phosphate levels (28). Decreased calcium levels were not influenced by treatment. Collectively, these findings denoted that R568 is a very efficient treatment of SHPT but is not capable of influencing mineral metabolism in uremic rats. One of the phases of AVC is infiltration of immune cells in the injured endothelium covering leaflets of the aortic valve. CD68 is a transmembrane glycoprotein that is highly expressed by human monocytes and tissue macrophages. CD68 presence was increased in uremic animals compared to controls, and R568 treatment had no influence on CD68 levels. Furthermore, increased levels of Runx2 were not affected by R568 treatment. As anticipated, following Runx2 increase, we found elevated levels of Osteopontin and Osteocalcin in uremic rats. No significant difference in Osteopontin and Osteocalcin levels between R568- and saline-treated rats was observed. Likewise, R568 treatment had no effect on osteoblast marker expression in aortic valves, and there were no significant changes in levels of Runx2, Osteopontin and Osteocalcin.

The progression of CKD causes SHPT as well as mineral and bone metabolism disease, and results in renal osteodystrophy and cardiovascular diseases. Recent studies have demonstrated the involvement of PTH in the development of calcified lesions, and strategies lowering PTH levels, including calcimimetics and parathyroidectomy, attenuate the progression of vascular calcification in CKD patients and uremic animal models (30–33). The major treatment for SHPT in CKD patients is calcimimetics, together with active vitamin D. In experimental studies, both calcimimetics – R568 and AMG641 – were shown to reduce high-serum PTH in uremic rats with SHPT without increasing serum calcium or phosphate, in contrast to active vitamin D sterols, thereby preventing the development of extraskeletal calcification (34). R568 was also shown to delay the progression of both aortic calcification and atherosclerosis in uremic apolipoprotein E-deficient mice (35). Similarly, the administration of R568 decreased calcitriol-induced aortic calcification in rats that underwent 5/6 nephrectomy (36) and R568 attenuated media calcification and proliferation of vascular smooth muscle and endothelial cells (37).

In chronic hemodialysis patients with SHPT, CINA treatment inhibits vascular and valvular calcification. In the ADVANCE trial, 360 hemodialysis patients were randomized to CINA or placebo and underwent baseline and follow-up computed tomography scans to assess coronary artery and aortic calcification (24). While the primary outcome, the Agatston score of coronary artery calcification, did not reach statistical significance (*p*=0.073), aortic and mitral valve calcification scores were significantly lower in the CINA group, and the progression of coronary artery calcification scores was slightly slower (24).

The disparity in our results may be attributed to the limited treatment duration, was sufficient to decrease PTH levels but insufficient to observe subsequent metabolic consequences. As previously shown (17), regression of AVC requires not only reduction of PTH levels but also decline in phosphate levels and increase of serum calcium. Our CKD animal model produced rapid-onset kidney disease with extensive tubule-interstitial fibrosis, tubular atrophy, crystal formation, and marked vessel calcification. Due to the rapid disease progression, it becomes challenging to observe the course of functional and structural damage in the cardiovascular system and the long-term implications of treatment approaches. To achieve improved outcomes, it is advisable to consider administering a combination of R568 with other medications, such as calcium supplements or phosphate binders.

In a study by Block et al., a total of 19,186 patients were enrolled; 5,976 of them received CINA and had 24-month follow-up. The study found a significant survival benefit with lower rates of adjusted cardiovascular mortality (HR,0.76; 95% CI,0.66-0.86) and all-cause mortality (HR,0.74; 95% CI,0.67-0.83) associated with CINA prescription in patients receiving intravenous active vitamin D analogue (20).

Meanwhile, recent studies revealed that suppression of PTH by CINA markedly attenuates vascular remodeling and calcification in uremia (21–24). CINA suppressed these calcification-related alterations by reducing serum PTH, calcium, phosphate, and the calcium-phosphorus product in a 5/6 nephrectomy rat model (23). Long-term CINA treatment reduced aortic calcification by more than 50% (21, 25). Moreover, studies in mice confirmed these results and reported delayed progression of atherosclerosis with CINA (21). Importantly, in all these animal studies (21–23, 25), medications were provided at the beginning of the study and, therefore, assessment the impact of the proposed intervention in preventing ectopic calcification but not in treating established calcification. Additionally, the treatment period in each of those animal studies lasted for six weeks or more, and CINA treatment had an effect on mineral metabolism. Additionally, the diversity of results may be explained by the complexity of the calcification process and varied pathways altered at different types of ectopic calcification.

### Limitations

In our CKD *in vivo* model, hyperphosphatemia and other metabolic abnormalities are introduced at the same time. Therefore, SHPT had a role in the development of calcification. Additionally, treatment duration was brief, which was sufficient to reduce PTH levels but not to lessen the calcification of the aortic valve. Our focus was solely on R568 treatment, without considering potential combination approaches. As mentioned earlier, solely reducing PTH levels is insufficient to reverse AVC. It imperative to concurrently lower serum phosphate levels to effectively address the calcification issue. Achieving this goal may involve a combination strategy such as using R568 in conjunction with phosphate binders, active vitamin D metabolites, or calcium supplements.

## Conclusions

Short-term R568 treatment effectively lowers serum PTH levels, however, it does not influence mineral metabolism, thus lacking an effect on AVC. The pivotal role of hyperphosphatemia and related metabolic changes in initiation of AVC and promoting osteoblast transformation in our experimental CKD model is evident. Additional studies are required to comprehensively assess the potential therapeutic strategies for managing AVC in the context of CKD.

## Data availability statement

The original contributions presented in the study are included in the article/supplementary material. Further inquiries can be directed to the corresponding author.

## Ethics statement

The animal study was approved by The Hebrew University Ethics Committee for the Care and Use of Laboratory Animals (permit no. 146944, approval number MD-15646-5). The study was conducted in accordance with the local legislation and institutional requirements.

## Author contributions

AA-S: Conceptualization, Data curation, Formal analysis, Investigation, Methodology, Validation, Visualization, Writing – original draft, Writing – review & editing. IG: Conceptualization, Data curation, Formal analysis, Investigation, Methodology, Software, Validation, Visualization, Writing – original draft, Writing – review & editing. SA: Conceptualization, Data curation, Formal analysis, Investigation, Methodology, Project administration, Supervision, Writing – review & editing. CL: Writing – review & editing. MG: Writing – review & editing. MS: Conceptualization, Data curation, Formal analysis, Funding acquisition, Investigation, Methodology, Project administration, Resources, Software, Supervision, Validation, Visualization, Writing – original draft, Writing – review & editing.
